# Viral Emerging Diseases: Challenges in Developing Vaccination Strategies

**DOI:** 10.3389/fimmu.2020.02130

**Published:** 2020-09-03

**Authors:** Maria Trovato, Rossella Sartorius, Luciana D’Apice, Roberta Manco, Piergiuseppe De Berardinis

**Affiliations:** Institute of Biochemistry and Cell Biology, National Research Council, Naples, Italy

**Keywords:** vaccines, emerging infectious diseases, viruses, epidemics, pandemics, antibody-dependent enhancement, SARS-CoV-2, COVID-19

## Abstract

In the last decades, a number of infectious viruses have emerged from wildlife or re-emerged, generating serious threats to the global health and to the economy worldwide. Ebola and Marburg hemorrhagic fevers, Lassa fever, Dengue fever, Yellow fever, West Nile fever, Zika, and Chikungunya vector-borne diseases, Swine flu, Severe acute respiratory syndrome (SARS), Middle East respiratory syndrome (MERS), and the recent Coronavirus disease 2019 (COVID-19) are examples of zoonoses that have spread throughout the globe with such a significant impact on public health that the scientific community has been called for a rapid intervention in preventing and treating emerging infections. Vaccination is probably the most effective tool in helping the immune system to activate protective responses against pathogens, reducing morbidity and mortality, as proven by historical records. Under health emergency conditions, new and alternative approaches in vaccine design and development are imperative for a rapid and massive vaccination coverage, to manage a disease outbreak and curtail the epidemic spread. This review gives an update on the current vaccination strategies for some of the emerging/re-emerging viruses, and discusses challenges and hurdles to overcome for developing efficacious vaccines against future pathogens.

## Introduction

Since the start of this century, a certain number of new or neglected pathogens have emerged from wildlife reservoirs and spilt over into human populations, causing severe diseases (1–3). Factors such as urbanization, globalization, travels, international commerce, aging, and climate changes have contributed to favor emergence, spread, and transmission of pathogens. Contacts among humans and potential zoonotic reservoirs are increasing, the number of travelers and their movements is growing, the aged population are more susceptible to infections, and the geographic distribution of pathogens within a previous endemic zone is changing (4, 5).

During the last decades, the global community faced several outbreaks of emerging and re-emerging infectious diseases, with high threats to the health security, biodefense, and economy worldwide (6, 7). The occurrence of significant disease outbreaks—such as SARS (severe acute respiratory syndrome) originating in China in 2002 (8), the 2009 H1N1 swine flu pandemic from Mexico (9), MERS (Middle East respiratory syndrome) that occurred in Saudi Arabia in 2012 (10), the West African outbreak of Ebola virus (EBOV) in late 2013 (11), the Zika virus (ZIKV) outbreak originating in Brazil in 2015 (12), the 2018 health emergence in Nigeria caused by Lassa virus (13), and the ongoing Coronavirus disease 2019 (COVID-19) pandemic (14)—has renewed interests in developing strategies to faster prevent, treat, and/or control emerging and re-emerging viruses with high epidemic potential. Usually, there is little or no knowledge about identity, epidemiology, and pathogenesis of a new infectious agent appearing for a first time in a certain geographic area (as in case of novel coronaviruses or new influenza variants), as well as the potential to spread out from the zoonotic reservoir, making hard to predict if, where, and when a disease outbreak will occur. The World Health Organization (WHO) and the National Institutes for Allergy and Infectious Diseases (NIAID) published a list of pathogens to be prioritized for research and development, given their epidemic potential. This non-exhaustive list comprises viruses, bacteria, protozoa, and fungi, causing diseases for which efficient countermeasures do not actually exist, or require new therapeutics (15, 16).

As proven by historical records, vaccination has played a pivotal role in reducing morbidity and mortality from devastating infectious diseases, successfully leading to disease eradication (i.e., smallpox), and generally decreasing infectious disease burdens. Even in presence of therapeutic options, vaccines are the valuable means to prevent infections and overall represent the much wanted achievement. However, even with worldwide efforts, getting a vaccine to the public takes time, and side effects, dosing issues, and manufacturing problems can all cause delays. Thus, we have to use this time with great concern. Generally speaking, in case of newly emergent diseases, conventional strategies might raise some issues. The unpredictable identity of largely unknown emerging pathogens, the lack of appropriate experimental animal models, and the time and costs for faster developing, producing, licensing, and globally distributing effective vaccine candidates are some of the major challenges to overcome in case of pandemic threats. Hence, new and/or alternative approaches in vaccine design and development are required to rapidly face outbreak situations (17).

This review will discuss the current vaccination strategies for some of the emerging and re-emerging viruses, as well as the approaches that might be suitable in face of global pandemic threats.

## Emerging and Re-Emerging Viral Infectious Diseases

Emerging and re-emerging pathogens represent a constant epidemic threat to humanity not only for the public health consequences but also for the economic, social, and political effects they may globally provoke. Therefore, a major public awareness and preparedness would be fundamental in fighting emerging infectious diseases. The terms “emerging and re-emerging infectious diseases” mainly refer to two major categories of infectious diseases: newly emergent infections, caused by novel pathogens; and re-emerging infectious diseases, caused by microbes reappearing after previous control, and/or eradication (1). Almost 60% of emerging infectious diseases are zoonoses, with the great majority of them originating in wildlife, and the number is constantly increasing. Climate changes have been related to the emergence of vector-borne diseases in severe environmental conditions, but this is a most debated issue, as well as the contribution of agricultural practices (18). In addition, the chances of infectious disease spreading could also include livestock/wildlife animal markets and consumption of those. A study where Australia was used as a model of urbanization has proposed a relation among increasing pandemic threats and urbanization: it ascribes the increased threat of pandemic to the high number of major city residents, the exponential intensification of international air traffic, and the commuter mobility network (19).

### Epidemic Versus Pandemic

Basically, an epidemic is an event that occurs when there is an increase, often sudden, in the frequency of a disease above what is normally expected in that population, in that area; while pandemic (from: παν = all, and δεμoσ = people) refers to an epidemic that spreads over several countries or continents at the same time, usually affecting a large number of people (20).

**FIGURE 1:**
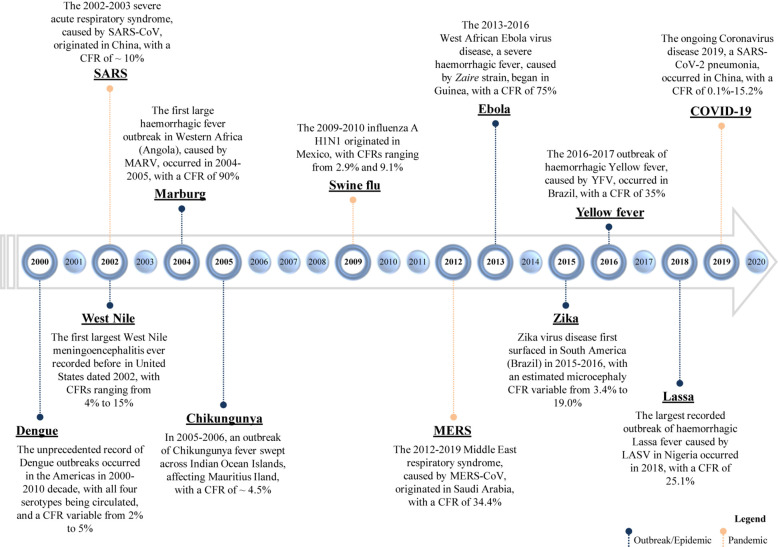
Timeline of emerging and re-emerging viral diseases. The year on the timeline is the year of the emergence or re-emergence of the schematically reported viral epidemic outbreaks within a certain geographic area; the overall given values of CFR (case fatality rate) refer to “the proportion of cases of a specified condition that are fatal within a specified time,” according to Dictionary of Epidemiology (228). SARS-CoV, severe acute respiratory syndrome coronavirus; SARS-CoV-2, severe acute respiratory syndrome coronavirus 2; MERS-CoV, Middle East respiratory syndrome coronavirus; MARV, Marburg virus; YFV, Yellow Fever Virus; and LASV, Lassa virus.

In the last decades, a certain number of viruses came to light for the first time or reappeared, giving rise to significant epidemics and pandemics (Figure 1 and Table 1).

**TABLE 1:** Emerging and re-emerging viral diseases.

**Disease**	**Virus**	**Family/Genus**	**Reservoir/spill-over hosts**	**Transmission**	**References**
West Nile fever (WNF)	WNV	Flaviviridae *Flavivirus*	Mosquitoes; birds/horses, dogs, rabbits	Mosquitoes	(22)
Zika fever	ZIKV	Flaviviridae *Flavivirus*	Mosquitoes; NHPs; domestic animals	Mosquitoes; vertical transmission	(23)
Yellow Fever (YF)	YFV	Flaviviridae *Flavivirus*	Mosquitoes; NHPs	Mosquitoes	(24)
Dengue fever (DF)	DENV	Flaviviridae *Flavivirus*	Mosquitoes; NHPs	Mosquitoes	(25)
Chikungunya fever	CHIKV	Togaviridae *Alphavirus*	Mosquitoes; NHPs	Mosquitoes	(25)
Lassa fever (LF)	LASV	Arenaviridae *Mammarenavirus*	Multimammate mouses	Rodent-to-human	(26)
Ebola virus disease (EVD)	EBOV	Filoviridae *Ebolavirus*	Fruit bats/NHPs; antelopes	Human-to-human	(27)
Marburg virus disease (MVD)	MARV	Filoviridae *Marburgvirus*	Bats/NHPs; humans	Human-to-human	(28)
Swine flu	A(H1N1)pdm09	Orthomyxoviridae *Influenzavirus A*	Pigs	Human-to-human	(29)
Severe acute respiratory syndrome (SARS)	SARS-CoV	Coronaviridae *Coronavirus*	Bats/palm civets	Human-to-human	(30)
Middle East respiratory sindrome (MERS)	MERS-CoV	Coronaviridae *Coronavirus*	Bats/dromedary camels	Human-to-human	(30)
Coronavirus disease 2019 (COVID-19)	SARS-CoV-2	Coronaviridae *Coronavirus*	Bats; likely malayan pangolins	Human-to-human	(31)

*NHPs, non-human primates.*

Epidemic outbreaks of viral diseases were mostly caused by flaviviruses generally transmitted by vectors, including West Nile virus (WNV) (21, 22), ZIKV (23, 24), Yellow Fever virus (YFV) (25, 26), and Dengue virus (DENV) (27, 28). Vector-borne diseases, including Chikungunya fever caused by Chikungunya alphavirus (CHIKV) (27, 29), are extremely difficult to eradicate because viruses are maintained in nature by propagation among vectors and hosts, without human–human contact. Moreover, dry and hot climate conditions seem to foster mosquitoes to bite humans than animals, increasing the risk of spreading diseases with a devastating impact (30). Today, most areas of the world are endemic for at least one flavivirus, with DENV being the most prevalent, and approximately 50–100 million people are infected each year. Among viral hemorrhagic fevers, Lassa fever (LF) is a rodent-borne acute disease caused by Lassa virus (LASV) (31), endemic in many West African countries, including Nigeria that experienced a high mortality rate in the 2018 outbreak (32). Ebola virus disease (EVD) and Marburg virus disease (MVD) are caused by members of the Filoviridae family, EBOV (33), and Marburg virus (MARV) (34), respectively. The 2013–2016 Ebola outbreak in West Africa was the largest since the virus was first discovered in 1976, with a case fatality rate for *Zaire ebolavirus* of 75% (35), while the largest recorded MVD outbreak occurred in Angola in 2004 (36).

Concerning pandemics, flu pandemics were reported three times during the twentieth century; genome analysis of pandemic influenza viruses dated 1918 (H1N1), 1957 (H2N2), and 1968 (H3N2) demonstrated that all viral strains fully or partially originated from non-human reservoirs, and that the ultimate origin of HA (hemagglutinin) genes are from avian influenza viruses (37), with the 1918 strain likely being the ancestor of the subsequent epidemic variants. Hence, the 1918 influenza pandemic has been called the mother of all pandemics (38). During the 2009 pandemic, caused by (H1N1)pdm09 virus, it has been estimated that 0.001–0.007% of the world’s population died of respiratory complications associated with the viral infection during the first 12 months, after the first reported case (39). The age of deceased people was below 65 years in almost 80% of cases, a peculiarity compared with the seasonal influenza epidemic. The mortality rates observed in 1968 and 1918 flu were of 0.03% and 1–3% due to H3N2 and H1N1, respectively, and ranging from 2.9 and 9.1% in 2009 (40). The 2009 H1N1 pandemic has been commonly referred to as swine flu for the swine origin of the virus, first isolated in Mexico and United States in April 2009. The viral genome sequencing indicated that the virus contains a combination of genes never reported in swine or humans before. It has been demonstrated that the swine has become a reservoir of H1 viruses with the potential to cause future pandemics (37). A (H1N1)pdm09 virus monovalent vaccine was produced in late 2009 (41), but the virus has not been eradicated and it continues to circulate as a seasonal variant, causing hospitalization, and death (42).

In November 2002, a first case of SARS was reported in Guangdong (China), and after 7 months, the coronavirus (CoV) causing the disease, named SARS-CoV, spread in 37 countries, giving rise to lower respiratory tract infections, with a poor outcome in 10% of cases (43, 44).

Middle East respiratory syndrome-CoV, the causative agent of MERS, was isolated in 2012. The coronavirus has caused isolated MERS outbreaks thereafter, becoming endemic in Arabian Peninsula, with a case fatality rate of 34.4% (43–45).

On March 11, 2020, WHO has declared the Coronavirus disease 2019 (COVID-19) outbreak a global pandemic. The disease is caused by a novel coronavirus, known as SARS-CoV-2, that shares almost 88% of the genome with that of SARS-CoV (46, 47). Actually more than 5.9 million (as of August 1, 2020) of people are infected, with an overall case fatality rate of 0.1–15.2% (48).

## Vaccine Platforms

In case of global public health emergencies, governmental and private organizations, vaccine developers, and regulatory authorities should all massively collaborate in selecting and funding the most suitable vaccine platform and strategy to quickly act and curtail disease outbreaks. At the outset of a disease outbreak, gaps in knowledge of identity, pathogenesis, epidemiology of the new emerging pathogen, time required to study the immune responses correlating with the outcome of the viral infection, and the lack of appropriate preclinical models susceptible to infection for testing a vaccine candidate pose several barriers and impediments to expedite vaccine design and development, and thus to ensure global vaccination coverage in time.

In the fight against newly emergent viruses, vaccine design might benefit from a range of platform technologies, including nucleic acid vaccines, viral-vector vaccines, and recombinant protein-based vaccines (likely to be administered with adjuvants) (17, 49). Compared with conventional vaccines, such as live attenuated and inactivated vaccines, molecular-based platforms might offer a more versatile tool against new emergent viruses, allowing a more fast, low-cost, and scalable vaccine manufacturing. Essentially, these platforms rely on the use of a system to deliver and present a new antigen (or a synthetic gene) to rapidly target an emergent pathogen. Theoretically, once a platform has previously met safety and efficacy requirements to be moved and advanced into the market, a candidate vaccine against a new virus might profit from the same system, production, and purification protocols, only replacing the disease target antigen (or inserted gene), thus streamlining the vaccine discovery.

### Inactivated and Live Attenuated Vaccines

**FIGURE 2:**
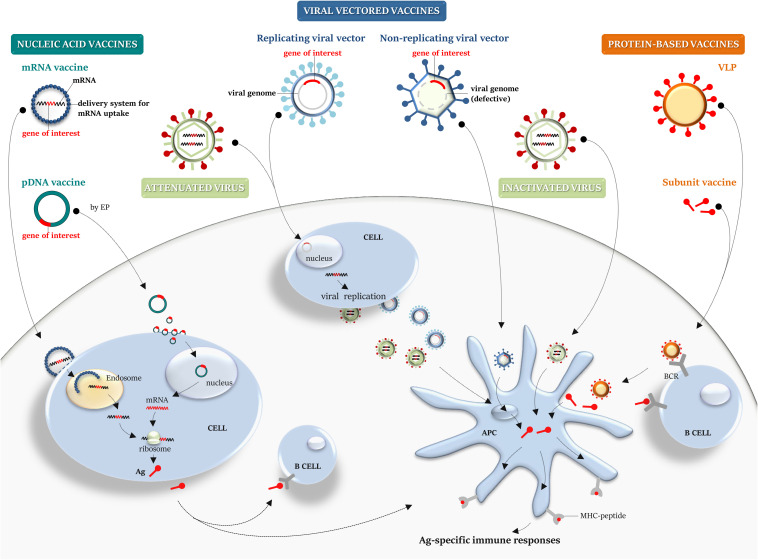
Platforms for vaccine manufacturing: a graphical overview. Nucleic acid, viral-vector, protein-based, live attenuated and inactivated vaccines are schematically illustrated. *Nucleic acid vaccines*: conventional non-replicating mRNA vaccine, containing the target gene sequence, can be encapsulated into a delivery system to aid its cellular uptake. Once released from endosome into the cytosol, it is translated by the host cellular machinery into the target antigen. A pDNA carrying a gene target reaches the nucleus to achieve transcription and translation into the cytosol. pDNA can be internalized by somatic cells (i.e., myocytes) and then the secreted antigen can be taken up by APCs or naïve B cell, priming immune responses. *Viral vectored vaccines*: defective viral vector, carrying a transgene cassette, can be employed as a system to deliver a transgene and allow the expression of the heterologous antigen within the infected cell. A recombinant replicating viral vector retains the ability to replicate and produce progeny virus particles that can then infect cells, leading to transgene expression and Ag processing and presentation. *Protein-based vaccines*: recombinant subunit vaccine or a VLP can be taken up by APCs for MHC presentation and B-cell recognition through BCR. *Virus vaccines*: compared with an inactivated virus, a live attenuated virus retains the ability to replicate and infect cells, mimicking the natural infection. APCs, antigen-presenting cells; MHC, major histocompatibility complex; Ag, antigen; pDNA, plasmid DNA; EP, electroporation; BCR, B-cell receptor; and VLP, virus-like particle.

In inactivated vaccine, the virus is rendered uninfectious using chemicals, such as formaldehyde or heat. This technology, conceived in the nineteenth century, is used for few vaccines still in use (i.e., inactivated polio, whole cell pertussis, and hepatitis A) (50). Live attenuated vaccines are obtained by passing the virus through animal or human cells until it picks up mutations that make it unable to cause the disease (i.e., measles, mumps, chickenpox, etc.); the attenuated smallpox was used for the massive vaccination campaign that successfully eradicated the infection (51), and currently, attenuated influenza viruses are used as vaccines against the seasonal influenza (52). The advantages of live attenuated vaccines are the intrinsic adjuvant properties, the ability to infect cells (Figure 2), and to activate the innate immune response. Interestingly, a safe SARS-CoV-2 inactivated vaccine (PiCOVacc) has been recently described as being able to induce specific neutralizing antibodies (NAbs) in experimental animal models (53), and a phase III clinical trial (NCT04456595) will soon assess efficacy and safety of this candidate in health care professionals (Table 2).

**TABLE 2:** Current vaccine platforms in clinical trials.

**Virus**	**Platform**	**Antigen**	**Vaccine**	**Phase**	**Trial N.**	**Sponsor**
WNV	Inactivated virus	Whole virus	HydroVax-001	I	NCT02337868	NIAID
	Viral vector	prM and E in YFV	ChimeriVax-WN02	II	NCT00442169	Sanofi
	pDNA	prM and E	VRC-WNVDNA017-00-VP	I	NCT00106769	NIAID
ZIKV	Inactivated virus	Whole virus	TAK-426	I	NCT03343626	Takeda
			ZPIV		NCT02937233	NIAID WRAI
			ZPIV		NCT02963909	NIAID WRAI
	Live attenuated virus	Whole virus	rZIKV/D4Δ30-713	I	NCT03611946	NIAID
	mRNA	prM and E	mRNA-1893	I	NCT04064905	Moderna
			mRNA-1325		NCT03014089	Moderna
	pDNA	prM and E	VRC-ZKADNA085-00-VP	I	NCT02840487	NIAID
			VRC-ZKADNA090-00-VP	II	NCT03110770	NIAID
	Viral vector	prM and E in MV	MV-ZIKA	I	NCT02996890	Themis Bioscience
	Viral vector	M and E in Ad.26	Ad26.ZIKV.001	I	NCT03356561	Janssen
DENV	Inactivated virus	Whole virus	TDENV-PIV	I/II	NCT02421367	GlaxoSmithKline
	Live attenuated virus	Whole virus	TDV	III	NCT02747927	Takeda
	Live attenuated virus	Whole virus	TetraVax-DV-TV003	III	NCT02406729	Butantan Institute
	pDNA	prM and E	D1ME100	I	NCT00290147	U.S. Army
	Viral vector	prM and E in YFV	Dengvaxia	III	NCT02993757	Sanofi
					NCT02948933	Sanofi
CHIKV	Live attenuated virus	Whole virus	VLA1553	I	NCT03382964	Valneva
	mRNA	prM and E	VAL-181388	I	NCT03325075	Moderna
	Viral vector	NC + E in MV	MV-CHIK	II	NCT02861586	Themis Bioscience
LASV	pDNA	GPC	INO-4500	I	NCT03805984	Inovio Pharmaceuticals
	Viral vector	GP and NP in MV	MV-LASV	I	NCT04055454	Themis Bioscience
MARV	pDNA	MARV/EBOV-GP	VRC-EBODNA023-00-VP	I	NCT00997607	NIAID
	Viral vector	MARV-GP in ChAd3	cAd3-Marburg	I	NCT03475056	NIAID
		MARV/EOBV-GP in MVA Multifilo + Ad.26	MVA-BN(R)-Filo + Ad26.ZEBOV	I	NCT02891980	NIAID
SARS-CoV	Inactivated virus	Whole virus	SARS-CoV	I	NCT00533741	NIAID
	pDNA	S	VRC-SRSDNA015-00-VP	I	NCT00099463	NIAID
MERS-CoV	pDNA	S	GLS-5300	I/II	NCT03721718	Inovio Pharmaceuticals
	Viral vector	S in ChAdOx1	ChAdOx1 MERS	I	NCT03399578	Oxford University
		S in MVA	MVA-MERS-S	I	NCT03615911	University Hamburg-Eppendorf
SARS-CoV-2	Inactivated virus	Whole virus	SARS-CoV-2	I/II	NCT04352608	Sinovac
				I/II	NCT04383574	Sinovac
				III	NCT04456595	Sinovac and Butantan Institute
	LNP-mRNA	S	mRNA-1273	I	NCT04283461	Moderna/NIAID
				II	NCT04405076	Moderna/NIAID
				III	NCT04470427	Moderna/NIAID
			CVnCoV	I	NCT04449276	CureVac AG
	pDNA	S	INO-4800	I	NCT04336410	Inovio Pharmaceuticals
				I/II	NCT04447781	Inovio Pharmaceuticals
			AG0301-COVID19	I/II	NCT04463472	AnGes, Inc.
			GX-19	I/II	NCT04445389	Genexine, Inc.
	Viral vector	S in Ad5	Ad5-nCoV	II	NCT04341389	CanSino Biologicals
				I	NCT04313127	Beijing Institute and CanSino Biologics
		S in ChAdOx1	ChAdOx1 nCoV-19	I/II	NCT04324606	Oxford University
	Subunit	RBD-dimer	Recombinant new CoV vaccine (CHO cells)	II	NCT04466085	Anhui Zhifei Longcom Biopharmaceutical
		S	SARS-CoV-2 rS	I/II	NCT04368988	Novavax
		RBD	KBP-COVID-19	I/II	NCT04473690	Kentucky BioProcessing, Inc.
	VLP	Coronavirus-Like Particle	CoVLP	I	NCT04450004	Medicago

*To retrieve the listed clinical trials, we visited the “https://clinicaltrials.gov” website and used the following keywords: virus name; biological; study phase (selecting the most advanced study); vaccine platform. prM, pre-membrane; E, envelope; NC, nucleocapsid; GPC, glycoprotein precursor; GP, glycoprotein; S, spike; RBD, receptor binding domain; MV, Measles virus; MVA, Modified vaccinia Ankara; rVSV, recombinant Vesicular Stomatitis Virus; Ad.26, Adenovirus type 26 vector; ChAd3, Chimpanzee adenovirus type 3 vector; VLP, virus-like particle; CHO, Chinese Hamster Ovary cells; LNP, lipid nanoparticles; NIAID, National Institute of Allergy and Infectious Diseases; and WRAI, Walter Reed Army Institute.*

### Nucleic Acid Vaccines: mRNA and DNA Vaccines

Nucleic acid vaccines include either mRNA or plasmid DNA (pDNA) vaccines (Figure 2).

Two types of mRNA vaccines were developed: conventional non-replicating mRNA vaccines and self-amplifying vaccines (or viral replicons). The *in* vitro enzymatic transcription (IVT) of a DNA template plasmid, containing the promoter sequence for the DNA-dependent RNA polymerase, provides a mature mRNA molecule, with the open reading frame that encodes the target antigen, the 5′ and 3′ flanking untranslated regions (UTRs), the 5′ cap, and the terminal poly(A) tail. Self-amplifying RNA (SAM) vaccines are commonly based on alphavirus genomes, where genes coding for the structural proteins are replaced with that encoding the target antigens, while the RNA replication machinery sequences are conserved, allowing intracellular antigen-encoding RNA amplification and higher antigen expression levels than the conventional mRNA vaccines (17, 54). Once the mRNA vaccine is delivered to the host cells and reaches the cytoplasm, it is translated *in vivo* by the host cellular machinery, providing the corresponding post-translationally modified antigen (Figure 2), thus mimicking the *in vivo* natural infection. mRNA vaccines activate the innate immune system, triggering host immune sensing receptors, and successively promoting adaptive immune responses (55). Several technological innovations have allowed to overcome some of the concerns associated with instability, half-life, inefficient *in vivo* delivery, and high innate immunogenicity of mRNA platform (56). mRNA vaccines do not produce infectious particles and potentially do not integrate into the host genome, reducing safety issues, and no anti-vector immunity is elicited. They can be quickly produced (likely within the time required to get genomic information from the new emergent virus), saving time and cutting costs. Thus, the mRNA platform offers a promising attractive alternative to conventional vaccines, should a disease outbreak occur.

No RNA vaccine has been yet licensed for humans, but encouraging results from preclinical and human clinical trials have shown that mRNA vaccines are able to induce safe and long-lasting immunity against different infectious viral diseases, including Zika (57), influenza (58–61), Ebola (61), Dengue (62), and other viral diseases (17). A SARS-CoV-2 mRNA-based vaccine entered clinical phases just 2 months after the identification of the viral genome sequence (NCT04283461), and a phase III study (NCT04470427) will assess its effectiveness to prevent COVID-19 (63–65). A clinical study (NCT04449276) is currently evaluating a similar vaccine in healthy adults (Table 2).

The DNA-based strategy, like the mRNA-based technology, offers a valuable platform to design and deliver any target of choice, due to safety profile, stability, ease of gene manipulation, and large-scale vaccine manufacturing, in short time at low costs. Thus, it might be a promising solution to overcome the hurdles of vaccine clinical development in the time a given unknown virus starts to spread in a certain area. A DNA vaccine is essentially based on a pDNA backbone with an inserted eukaryotic expression cassette. A pDNA can be used to encode viral antigens, which can lead to antigen-specific immune responses (66, 67), on cellular uptake and *in vivo* long-term gene expression, potentially providing advantages over mRNA vaccines in terms of protein coding capacity, and amount and extent of antigen production. Unlike mRNA, pDNA needs to cross both plasma and nuclear membranes to enter into a cell target, reach the nucleus, and achieve transcription (Figure 2). Advances in pDNA delivery devices (i.e., use of gene gun; *in vivo* electroporation, EP), and delivery systems (i.e., encapsulation in LNPs; adsorption to polymers), have greatly enhanced molecular stability, delivery efficiency, uptake, and antigen expression. In addition, the use of optimized pDNA formulations and encoding molecular adjuvants, to be administered in prime-boost strategies or simultaneously with other vaccine platforms, has generally improved the low protective immune-stimulatory profile of pDNA (67). However, some potential safety concerns should be considered, including long-term persistence upon administration, which could eventually lead to genomic integration events, antibodies against bacteria-derived plasmids that could potentially trigger autoimmune diseases, and unwanted side effects due to encoded and co-delivered molecular adjuvants (17, 67).

Even though no DNA vaccine has been yet licensed for use in humans (four for veterinary use), this platform has shown great promise for several emerging viral diseases, including Ebola and Marburg (68), MERS (69), West Nile (70), Dengue (71), Chikungunya (72), and other viral diseases (17), and more recently for COVID-19 (73). Currently, DNA-based vaccine candidates, encoding the S protein from SARS-CoV-2, have moved into clinical phase I/II development (63, 65, 74) (Table 2).

### Viral-Vector Vaccines

Recombinant viral vector-based platform employs either live replicating often attenuated or non-replicating viruses as vector vaccines (Figure 2). Viral vector vaccines represent the biotechnological evolution of live attenuated and inactivated vaccines: a viral backbone devoid of the replication machinery to be used as a shuttle to express *in vivo* the chosen target antigen. Several viral backbones have been exploited to generate viral-vector vaccines. Targeted deletion of replication genes represents the non-empirical way of virus attenuation, allowing the generation of a wide array of viral vectors, engineered by insertion of a transgene cassette.

The modified virus Ankara (MVA) is an attenuated form of the Vaccinia Virus (VACV), derived from more than 570 passages in chick embryo fibroblasts, a method that empirically modifies the viral genome, without affecting the immunogenicity (75). It is able to infect multiple cell types but cannot replicate inside the infected cells, ruling out the safety concerns related to the use of live vaccines.

One of the drawbacks in the use of a viral vector vaccine is that multiple immunizations lead to the host response against the structural viral proteins, limiting the efficacy of vaccination, as demonstrated in a study based on cellular immune response. To overcome this limitation, the heterologous prime-boost regimen has been introduced in several clinical trials, where two different viral vectors or a pDNA prime-viral vector boost were tested (76). Risks of integration into the host genome do potentially exist, as some viral vectors enter to the nucleus of cells to achieve transcription and replication. A major restrain in the production of viral vector vaccines is the time-consuming manufacturing; several attempts to accelerate vaccine production are in development, like selecting cell lines with higher yield or choosing the best promoter for transgene expression to reduce vaccine doses (77).

Among the available viral vectors, the adenoviruses are the most used in priming the immune response, being able to induce humoral and cellular responses (78). A pre-existing anti-vector immune response jeopardized the vaccine response in adenoviral-based clinical trials (79). To avoid pre-existing immunity, adenoviral vectors of non-human origin or rare serotypes have been used as vaccine platform. The use of chimpanzee adenoviral vectors proved to be safe and effective in clinical trials conducted against Ebola (80) and Respiratory Syncytial Virus (RSV) (81). Vesicular Stomatitis Virus (VSV), a single-stranded negative sense RNA virus that naturally infects livestock, represents an attractive safe alternative over other viral vectors due to low risk of pre-existing immunity, lack of DNA molecules during replication, and ability of VSV-based vaccines to induce effective humoral responses (82).

For humans, two viral-vector vaccines are available: Imojev, a Japanese encephalitic virus (JEV) vaccine; and Dengvaxia, a Dengue vaccine (both from Sanofi Pasteur). Both are produced using the chimeric YFV as vector: two of the YFV genes have been replaced by genes encoding the pre-membrane (prM) and the envelope (E) protein of JEV or DENV, and the chimeric viruses are propagated in cell culture (83, 84). Conversely, several viral vector vaccines have been licensed for veterinary use because of the less stringent regulatory requirements (76). To face COVID-19, an Adenovirus type 5 vector expressing SARS-CoV-2 S protein (Ad5-nCoV) has been advanced into phase II trial (NCT04341389), while a phase III study (ISRCTN89951424) is currently investigating the chimpanzee adenoviral vector (ChAdOx1 nCoV-19), expressing the same protein (63, 65, 74) (Table 2).

### Recombinant Protein-Based Vaccines

Recombinant protein-based vaccines consist of immunogenic proteins from the target pathogen. Once identified, recombinant proteins can be produced on a large scale, in bioreactors, using heterologous expression systems, like bacteria, yeast, plants, insect, or mammalian cell lines, depending on the post-transcriptional pattern of modification required (85). Vaccines based on recombinant proteins represent a safe platform because they do not contain pathogen-derived genetic information, and the manufacturing does not require manipulation of live pathogens. They might represent a platform of choice when a fast response to an epidemic is on demand, as the vaccine production can start once the genome of the new virus has been sequenced, even before the virus isolation.

Protein-based vaccines can be obtained producing recombinant virus subunits (SUVs) that can be administered in combination with adjuvants to improve the host immune response against the recombinant viral antigens (86). Recombinant proteins derived from viral capsid can self-assemble into virus-like particles (VLPs), high ordered and repetitive structures devoid of the viral genome. VLPs display antigenic epitopes in their original conformation in high copy number, they retain the size and geometrical organization of the original virus (mainly icosahedral or rod shape), preserving the viral immunogenicity due to the ability to crosslink B cell receptor on B cell surface (87), and to be taken up by antigen-presenting cells (APCs) (88, 89) (Figure 2). Several strategies have been proposed to improve dendritic cell (DC) uptake, by expressing targeting molecules such as antibodies directed against endocytic receptors, and to augment immunogenicity, through simultaneous delivery of maturation stimuli, like TLR agonists (90, 91). When not able to self-assemble into a VLP, the selected antigen can be expressed as chimeric protein: several VLP platforms are available for the display of heterologous antigens on the viral coat proteins. Recombinant VLPs from plant virus, like Tobacco mosaic virus (92), or alpha mosaic virus (93), are easily produced, competing for speed and cost of production with VLP platform based on mammalian viruses (94). The most used VLP platform is the HBcAg-VLP, the core antigen from hepatitis B virus (HBV) (95). It is also possible to chemically attach the heterologous antigen to a preformed VLP by using conjugation methods (96). Although this strategy could increase the manufacturing costs, it might be suitable when the expression of recombinant antigens affects the VLP assembly.

To date, VLP-based vaccines that have been licensed for human use include Cervarix (Merck & Co., Inc.) and Gardasil (GlaxoSmithKline Biologicals), used in prophylaxis against human papilloma virus (HPV), formed by the L1 major viral capsid protein; Engerix-B (GlaxoSmithKline Biologicals) and Recombivax-HB (Merck & Co., Inc.), consisting of HBV surface antigen (HBsAg), with a lipoprotein-like structure; and Flucelvax (Flucelvax Tetra in EU and Flucelvax Quadrivalent in United States), consisting of surface antigens from four influenza strains, recommended for individuals at high risk. A recombinant hepatitis E virus (HEV) vaccine, named Hecolin HEV 239 (Xiamen Innovax Biotech Co., Ltd.), containing the capsid protein from genotype 1 Chinese viral strain, has been licensed for use in China.

Currently, several recombinant protein-based vaccines against SARS-CoV-2 are under preclinical and clinical evaluation (64, 74) (Table 2). It is worth mentioning that Kim and colleagues designed and developed a SARS-CoV-2 subunit vaccine within 4 weeks of the identification of SARS-CoV-2 S protein N-terminal domain S1 sequence. Delivery of recombinant subunit vaccines by microneedle array resulted in potent antibody response in mice (97), and vaccination with a SARS-CoV-2 Spike S1-Fc fusion protein induced antibody responses in small animal models and NAbs in monkeys (98).

## Vaccines for Viral Infectious Diseases: State of Art

In Table 2 are listed the vaccine candidates that currently moved into clinical trials for preventing the viral infectious diseases discussed in the following section.

### WNV

West Nile virus includes five lineages; among them, lineage 1 was classified as the most virulent, while lineage 2 is considered more attenuated. However, during a serious outbreak in Hungary in 2008, the sequencing of lineage 2 showed some genetic mutations that demonstrated the increased virulence of this strain and its explosion throughout the central Europe (99, 100), causing renewed interest in the development of a vaccine against WNV. 20 years after the epidemic that hit the United States, no WNV vaccine has been yet released for human use, while four vaccine formulations are on the market for veterinary use, three based on the whole inactivated virus (WN Innovator, Vetera WNV, and Prestige WNV), and one on recombinant vaccine expressing WNV prM/E into a canarypox backbone (Recombitek Equine WNV) (101, 102). These vaccines completely protect horses from viral infection but require subsequent administrations and several booster doses overtime.

For the development of a vaccine for humans, many different platforms were used in preclinical studies, and many of them entered into phase I/II trials, including hydrogen peroxide–inactivated whole virus (HydroVax) vaccine (NCT02337868) (103), a recombinant truncated form of WNV E protein (104), recombinant chimeric live attenuated viral vectors, employing YFV (105), or MVA (106) delivering WNV prM/E proteins (NCT00442169), pDNA vaccines encoding prM/E (NCT00106769) (70, 107). All the Envelope-based vaccines induced NAbs against both WNV lineages 1 and 2, but some candidates are unable to generate long-lasting antibody responses, requiring multiple administrations (103, 108, 109). Thus, further improvements are needed for the development of next-generation vaccines (110). Recently, a WNV replication-deficient vaccine candidate with a deletion of the non-structural protein NS1 has been shown to protect mice from a highly lethal viral challenge, after a single dose, without adverse effects (111).

### ZIKV

During the 2015 outbreak in Brazil, an abnormal microcephaly number and other birth defects in newborns were reported (112). For this reason, vaccination of pregnant and of reproductive-age women became an urgency. Shan and colleagues developed a candidate vaccine, using a live attenuated viral strain containing a deletion in the 3′ region of the virus genome. This vaccine induced strong and protective antibody response, after a single injection in mice and macaques, and reduced viral RNA in placental and fetal tissues in infected mice (113). The immunized mice also developed a robust T-cell response (114). Although promising, this attenuated virus-based formulation does not meet the safety standards required to be used to vaccinate pregnant women, whose prophylaxis requires a vaccine that fulfill higher safety standards.

A number of different replication-deficient viral vectors have been recently developed and are currently under evaluation. Immunization of mice with a vaccine based on MVA delivering the ZIKV prM and the E structural proteins (MVA-ZIKV) elicited NAbs and potent ZIKV-specific CD8^+^ T-cell responses, mainly with an effector memory phenotype (115). A rhesus adenovirus serotype 52 vector (RhAd52), expressing ZIKV prM and E proteins, induced high titer of ZIKV-specific antibodies after the first prime, offering complete protection against subcutaneous ZIKV challenge, in mice (116), and rhesus monkeys (117). These adenoviral-based vaccines induced antibodies that were also maternally transmitted (118). In addition, Abbink et al. using the rhesus macaque model demonstrated that a complete anti-ZIKV immunity can only be achieved through vaccination with a combination of different vaccine platforms (117). ZIKV vaccine candidates currently in phase I clinical trials include inactivated and live attenuated vaccines, mRNA and pDNA vaccines, and recombinant viral-vectored vaccines, mainly targeting the prM and E proteins (119). A DNA-based vaccine encoding the prM signal sequence from JEV and ZIKV E proteins moved into phase II (NCT03110770), showing immunogenicity and safety in humans (120).

### YFV

A protective and efficacious vaccine against YFV is currently available. To date, the main type of YF vaccine produced on a large scale is based on the live attenuated 17D virus vaccine. This vaccine is obtained after numerous passages of Asibi virus strain in mouse and chicken embryo that generate a strain with accumulated mutations in the envelope protein. These mutations affect the virus binding to the host receptor, reducing its neurotropism and vicerotropism, and mosquito transmissibility (121). Because the vaccine is produced in chicken embryo, there are issues related to manufacturing costs and vaccine availability. The interruption of vaccination coverage against YF in endemic countries has caused major outbreaks in Africa and South America in 2015 and 2016, which exhausted the 17D vaccine stockpiles leading to the use of an emergency “fractional dose” campaign in the Democratic Republic of Congo (122). Thus, the fluctuating demand for doses during outbreaks makes the accessibility to the vaccine still a problem to be solved.

### DENV

The need for a vaccine against DENV has become an urgency only in recent decades. Dengue fever is caused by four distinct virus serotypes, DENV1–4, able to circulate simultaneously in endemic areas, making extremely difficult the development of a broad protective vaccine. Recently, the Food and Drug Administration approved the first Dengue vaccine by Sanofi-Pasteur, named CYD-TDV or Dengvaxia (123, 124), a tetravalent live attenuated virus vaccine on YFV backbone, whose release has generated controversy due to evidence that the administration can increase the risk of a more severe form of the illness in people with a pre-existing immunity toward other DENV strains (125, 126). For this reason, the use of Dengvaxia is strictly limited, depending on age (between 9 and 16) and serostatus of recipients to vaccinate (exclusively individuals who had a previous DENV infection), generating concerns about its cost–benefit balance. Studies for the development of a safer vaccine are still ongoing, and candidate vaccines include a tetravalent Dengue purified inactivated virus vaccine, currently in phase I/II clinical trial (NCT02421367), and two live attenuated tetravalent chimeric TDV (DENVax), and TVD 003/005 (TetraVax-DV) vaccines, currently in phase III clinical trials (NCT02747927; NCT02406729) (127).

### CHIKV

No vaccine is actually available to prevent CHIKV infection. Among the candidates in ongoing studies, two of them achieved and completed phase I or II trials: VLA1553 and MV-CHIK vaccines. VLA1553 candidate (by Valneva) is a live CHIKV (La Réunion isolate LR2006 OPY1) attenuated by a partial deletion of the gene encoding the non-structural replicase complex protein. This vaccine induced immunity lasting over 20 months after a single shot immunization (NCT03382964). MV-CHIK vaccine is a live attenuated measles-vectored CHIKV vaccine that induced CHIKV-specific NAbs and shown to be well tolerated by all the participants (NCT02861586) (128). Recently, Moderna Therapeutics tested a vaccine based on engineered mRNA encoding CHIKV structural polyproteins (mRNA-1388) in a phase I clinical trial. As shown in preclinical studies, this formulation induced strong immune responses after one single injection, totally protecting mice from developing the disease (129).

### LASV

Currently, there is no vaccine for LASV infection. Among the difficulties to tackle in the development of effective vaccines, there are the high genetic diversity of LASV strains and the absence of established correlates of protection. The high titer of antibodies does not prevent the viral replication, suggesting that protection to LASV is probably cell mediated (130). Vaccine platforms under advanced development include a DNA-based vaccine (INO-4500 from Inovio), moved into phase I (NCT03805984) (131), and a live attenuated vaccine based on measles virus, expressing LASV glycoprotein and nucleoprotein (MV-LASV). The MV-LASV vaccine gave promising results in preclinical animal models, being able to activate innate immunity, adaptive T-cell and B-cell responses (132), and it has been advanced to phase I clinical trial (NCT04055454), aimed at evaluating the optimal dose.

### EBOV

Although the identification of EBOV dates back to 1976 (133), only few studies on vaccine candidates and four clinical trials were conducted before the West African outbreak in late 2013. Vaccines against Ebola virus have been extensively reviewed previously (134–136).

### MARV

Several vaccine platforms have been tested in preclinical animal models and shown to be able to protect animals from MARV infection and to induce both humoral and cellular immune responses. These include VLPs (137), DNA vaccines (68), recombinant adenoviral vectors (138), and rVSV (139, 140). Many works have emphasized the use of a multivalent vaccine formulation to achieve protection against different filoviruses. Vaccination with a single dose of a trivalent formulation based on rVSV expressing glycoproteins from EBOV, *Sudan ebolavirus* (SUDV), and the Angola strain of MARV elicited antibodies specific for the three glycoproteins in non-human primates (NHPs) and a balanced T-cell response sufficient to protect against the viral challenges (141). Similarly, VLPs delivering a trimeric hybrid glycoprotein from MARV, EBOV, and SUDV fully protected vaccinated animals from MARV challenge, inducing specific NAbs (142). Using an enhanced DNA-based platform encoding the envelope glycoprotein from MARV and EBOV, Shedlock and colleagues showed that a polyvalent-filoviral vaccine candidate, delivered by *in vivo* EP, elicited in preclinical models robust NAbs and cytotoxic T cells, completely protecting animals from the viral challenge, after a single dose administration (68). Actually, a multivalent phase I study (NCT02891980) is evaluating safety and immunogenicity of two heterologous and two homologous prime-boost regimens using a MVA multi-filo and Ad26 Zaire Ebola (Ad26.ZEBOV) vaccines (143) in healthy volunteers, with the aim to analyze the protective response to different filoviruses.

## Current Status on Coronavirus Diseases

Coronaviruses are a group of single-stranded RNA viruses that have been present in humans for at least 500–800 years and all originated in bats (144, 145). Earlier than 2019, six coronaviruses had been known to cause diseases in humans: HCoV-229E, HCoV-043, HCoV-Nl63, HCoV-HKN1, SARS coronavirus (SARS-CoV), and MERS coronavirus (MERS-CoV) (146). In late 2019 and early 2020, a novel coronavirus was discovered to be the cause of a rapidly spreading outbreak of respiratory disease, including potentially fatal pneumonia, in Wuhan, China. The virus, provisionally designated 2019-nCoV and later given the official name SARS-CoV-2, owing to its similarity to SARS-CoV (then named SARS-CoV-1), was isolated and the viral genome sequenced. SARS-CoV-2 was characterized as a beta-coronavirus (147). The disease caused by the virus was officially named Coronavirus Disease 2019 (COVID-19) by WHO.

Coronaviruses are capable of adapting quickly to new hosts through the processes of genetic recombination and mutation *in vivo*. Point mutations alone are not sufficient to create a new virus. However, this may occur when the same host is simultaneously infected with two coronavirus strains, enabling recombination of genomic fragments of hundreds or thousands of base pairs long and thus making a new virus (148, 149). This susceptibility enabled the emergence, in approximately two decades, of three new human coronavirus species with epidemic potential: SARS-CoV-1, MERS-CoV, and SARS-CoV-2. Coronaviruses enter cells *via* binding to a host receptor followed by membrane fusion. The angiotensin-converting enzyme 2 (ACE2) was identified as the cell receptor for SARS-CoV (150), and recently also for the new SARS-CoV-2 (151), while MERS-CoV binds the dipeptidyl peptidase 4 (DPP4) receptor, also known as CD26 (152). The S protein is used for virus–cell receptor interaction during viral entry (153). Transmission of the virus during the viremic stage of disease is primarily *via* respiratory secretions (droplets) or direct contact. SARS-CoV-2 is extremely contagious, with an estimated basic reproduction number (R0) of 2.24–3.58 (154). In contrast, the R0 for both SARS-CoV-1 and MERS-CoV is less than 1 (155). It soon became apparent that infected individuals might be capable of transmitting the virus during the prodromal period (156).

### Prevention

Social distancing strategies (quarantine and community containment) represent the only efficacious means of controlling coronavirus spread in the absence of effective drugs or vaccine against the pathogens. Of importance, for preventing the spread of the disease caused by contact with patients or contaminated fomites, hygiene measures are also mandatory, such as washing hands with soap and water or with alcohol-based preparations. Indeed, coronaviruses are able to survive on various surfaces for few days but can be inactivated by disinfection (157). Finally, because it has been demonstrated that the overlap between human and animal ecosystems have given to coronaviruses the opportunity to cross the species barrier, to prevent future zoonotic diseases, a coordination with veterinary experts as well as stricter laws governing the trade of wild animals would be necessary.

### Vaccines

Humans are extremely exposed to these pathogens because these viruses had not previously circulated in the human population, as testified by the absence of antibodies against coronavirus in healthy people. In addition, the innate immune response has demonstrated to be insufficient in controlling coronavirus infection because decreases in viral load are coincident with the specific antibody response (158, 159). In this context, vaccines represent a much expected resource. A hopeful premise is represented by the successful containment of coronavirus epidemics in farm animals by vaccines, based on either killed or attenuated virus (160), and concerning SARS-CoV-2 by the finding that specific antibodies are detectable in 100% of patients with COVID-19, 17–19 days after symptom onset (161), and that the magnitude of antibody titers positively correlated with viral neutralization potency (162).

After the SARS outbreak, several vaccines were formulated based on various strategies, as recombinant S protein-based vaccines, attenuated and whole inactivated vaccines, as well as vectored vaccines. Pre-clinical data showed animal protection from challenge with SARS-CoV-1. However, sterilizing immunity was not always achieved (163). In few cases, the use of live virus as a vaccine resulted in complication including lung damage, eosinophil infiltration, and liver damage in animal models. Moreover, a study of vaccination with inactivated SARS-CoV-1 in NHPs reported enhancement of disease caused by specific epitopes on the S protein [reviewed in (64)]. Another issue is related to the length of a protective immune response. Both humoral and cellular responses have been found important for lasting protection. In long-term studies of recovered SARS patients, antibody responses waned after approximately 6 years, while T-cell responses persisted, suggesting that the latter is required for long-lasting immunity.

Concerning MERS-CoV, the vaccines proposed target the S protein (164–166), including mucosal vaccine for intranasal administration (167). However, cases of enhanced lung diseases were also reported in preclinical models of vaccination in mice (168). New MERS-CoV vaccines in development also include live attenuated, protein subunit, and DNA vaccines (169, 170). Recently, a small animal model that replicates MERS-CoV transmission has been developed (170) and will help the pre-clinical studies.

Following the alarming data and casualties provoked by COVID-19, a strong effort by the research community is going on at the moment, and WHO has been informed of dozens of vaccines in preparation using different platforms, as mentioned in section “Vaccine Platforms.” Some of these candidate vaccines are already in phase I/II clinical trials, while others have been advanced to phase III studies (63, 65, 74) (Table 2). However, it is possible that a SARS-CoV-2 vaccine will not be available for another 12–18 months. Recently, a rhesus macaque model that recapitulates SARS-CoV-2 infection has been developed to study immunopathogenesis and test vaccine candidates (73, 171).

### Passive Immunotherapy

Therapy based on passive administration of anti-coronavirus antibodies, isolated from patient sera, also represents a much wanted option for the treatment of coronavirus diseases (172), and a global effort is pursued in this direction to treat patients before the achievement of a validated vaccine. In addition, researchers are trying to produce in laboratory specific and protective anti-coronavirus antibodies. In the case of SARS outbreak, a monoclonal antibody (MAb) with neutralizing activity, being able to block receptor association, was identified and described (173). Moreover, neutralizing MAbs have also been produced to fight MERS-CoV infection. In a collaborative study by US and Chinese researchers, MAbs targeting the receptor (CD26/DPP4) binding domain of MERS-CoV spike glycoprotein were reported (174). Japanese researchers have also investigated anti-CD26 MAb for MERS-CoV and have identified the humanized MAb YS110 as a promising candidate (175). Finally, in the case of SARS-CoV-2 outbreak, Dutch researchers claimed the identification of a human MAb named 47D11 able to block SARS-CoV-2 infection (176). Recently, a MAb able to cross-neutralize SARS-CoV-2 has been identified from memory B cells of a SARS-CoV-infected individual. The antibody, named S309, engages the S receptor-binding domain, recognizing a highly conserved protein/glycan epitope distinct from the receptor-binding motif (177). More recently, other potent neutralizing antibodies were isolated by different research institutions (178–180).

Amidst the gamut of high-affinity antibodies with the potential to neutralize human pathogenic viruses, single-domain antibodies, referred to as nanobodies or Nbs (15 kDa), and nanobody-based human heavy chain antibodies (75 kDa) derived from camelids might be harnessed as useful therapeutics for the ongoing COVID-19 pandemic (181). Camelid heavy-chain-only antibodies (HCAbs) are composed of two heavy chains with a single variable domain (VHH) as the target-binding module. Recombinant VHHs, devoid of the effector domains, act as single-domain antibodies and harbor advantageous features over conventional antibodies (higher thermal and chemical stability, higher solubility, smaller size, lower susceptibility to steric hindrances, ease of manufacturing, and simple structure) to have been recently proposed as prospective therapeutic candidates against various infectious pathogens (181). VHHs isolated from a llama subcutaneously immunized with perfusion-stabilized SARS-CoV-1 and MERS-CoV S proteins have been recently characterized and shown to be able to neutralize S pseudotyped viruses *in vitro*, interfering with the host cell receptor binding (182). Interestingly, SARS-CoV-1 S-directed VHH cross-reacted with SARS-CoV-2 Receptor Binding Domain (RBD) and neutralized SARS-CoV-2 S pseudoviruses *in vitro* as a bivalent human IgG Fc-fusion format, underscoring the potential of VHHs to treat coronavirus diseases (182).

## Vaccine Hurdles: Flavivirus Cross-Reactivity and Antibody-Dependent Enhancement

### Flavivirus Cross-Reactivity

Because of the global spread of diseases caused by flaviviruses, understanding the cross-reactivity of anti-viral immunity among these viruses is of crucial importance for predicting the evolution of viral disease outbreaks.

Recently, the analysis of PBMCs isolated from individuals infected by DENV or vaccinated with DENV TV005 or YF17D vaccines, and pulsed with a pool of antigens from autologous and heterologous flaviviruses, indicated that both CD4 and CD8 T-cell responses were specific, with little or no cross-reactivity, despite the high level of homology (183). Individuals pre-exposed to DENV infection developed T-cell responses against non-structural ZIKA proteins rather than structural envelope protein, suggesting that previous flaviviral infections biased the T-cell response toward more cross-reactive non-structural epitopes (184). Studies enrolling mothers who gave birth to microcephalic babies after ZIKV infection, showed serological evidence of a pre-existing anti-Dengue response, suggesting that vaccination against DENV does not protect against ZIKV microcephaly (185). However, cross-reactive antibodies between ZIKV and DENV have been described, mainly targeting the structural dimeric envelope protein (186, 187). The antigenic sequences are both linear and quaternary, with NAbs mainly recognizing the latter. The high-conserved E protein fusion loop induces cross-reactive but weak NAbs that can be a marker of worst outcome during subsequent flaviviral infections (188). A research concerning ZIKV-specific B-cell responses in three DENV-experienced donors showed that 5 months after the infection, the pool of antibodies comprised both poorly NAbs derived from pre-existing DENV-induced memory B cells, associated with an enhanced ZIKV infection *in vitro*, and potent ZIKV-specific antibodies originated *de novo* (189, 190). The possibility that WNV-specific antibodies may drive the infection by other flaviviruses is still controversial, even if cross-reactivity was demonstrated. Plasma samples from convalescent human WNV patients were shown to enhance ZIKV infections by antibody-dependent enhancement (ADE) phenomenon (191); conversely, mice previously infected with ZIKV and challenged with WNV showed enhanced protection toward the second infection (192).

The immunological Flavivirus cross-reactivity, the ADE phenomenon (discussed below), genetic mutations that increase the virulence, potential pre-existing immunity concerns, combined with the necessity to increase cost-effectiveness of marketable products are among the issues that have limited the development of successful vaccines until now. The use of T-cell inducing vaccines or proteins with mutations into conserved Envelope fusion-loop epitopes might be useful to overcome the cross-reactivity hurdle (193).

### ADE: Antibody-Dependent Enhancement

Known as ADE of viral infection, ADE is a phenomenon occurring when antibodies facilitate virus entry into the host cells, driving viral replication and increasing infectivity, with subsequent severe outcomes.

**FIGURE 3:**
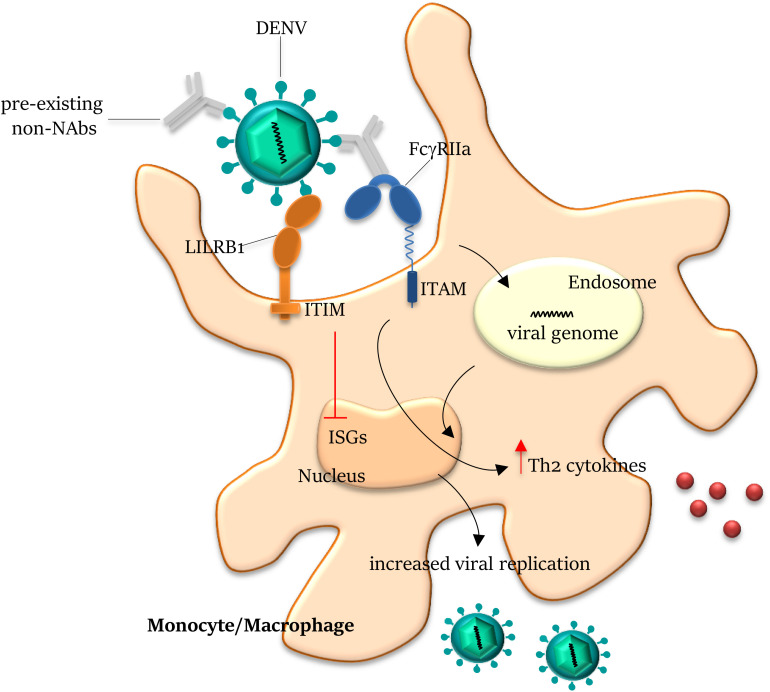
Antibody-dependent enhancement on Dengue infection. Antibodies generated from a previous DENV infection can recognize but do not neutralize another DENV serotype and can lead to antibody-dependent enhancement (ADE) of entry of the latter virus into host cells. The pre-existing non- (or sub-) neutralizing antibodies bind DENV through the Fab domains and mediate viral entry into FcγR-expressing cells. On engagement by the Fc domains, the virus–antibody immune complex is internalized by the activating FcγRIIa within the endosome. Co-ligation of FcγRIIa and LILRB1 (leukocyte immunoglobulin-like receptor-B1) to opsonized DENV drives the inhibitory signal cascade *via* immunoreceptor tyrosine-based inhibition motif (ITIM) pathway, abrogating the expression of ISGs (Interferon Stimulated Genes). Ligation of FcγRIIa to immune complex also increases Th2 cytokine production and reduces IFNγ, inhibiting the JAK/STAT signaling pathway, overall resulting in the suppression of the antiviral response and increase of viral replication. NAbs, neutralizing antibodies; and ITAM, immunoreceptor tyrosine-based activation motif.

Among the several stumbling blocks in realizing a safe vaccine, ADE is a phenomenon largely underestimated, but that can produce severe adverse effects, rendering vaccinated individuals more predisposed to develop harsh symptoms after infection (194). The first report of ADE dates 1964 (195). The molecular mechanisms disclosed the involvement of FcγR (196) and complement receptors (197). When an antiviral antibody (induced by vaccination or viral infection) with no neutralizing or sub-neutralizing activity is produced, it can act like a bridge between the virus and the FcγR expressed on the surface of immune cells, leading to viral uptake (Figure 3), as demonstrated for DENV, ZIKV, WNV, Influenza, SARS-CoV, MERS-CoV, and EBOV (194). The role of complement receptor has been demonstrated in EBOV response: two antibodies directed against epitopes in close proximity bind the C1q, forming an immune complex able to enhance the virus entry into a target cell (198), whereas in an animal model of MERS-CoV, C3a and C9 protein level increase was observed after passive immunization (199). The first licensed vaccine against DENV (CYD-TDV-Dengvaxia) caused hospitalizations in two large multicenter phase III trials; after result revision, it has been estimated that in seronegative individuals, it can produce adverse effects (194), and WHO recommendations are to vaccinate only seropositive individuals in endemic areas of age older than 9 years. Using a mathematical model of DENV transmission to formulate hypothesis on vaccine trial results, it was speculated that “Seronegative recipients gain transient protective cross-reactive immunity akin to that observed for natural infection,” increasing the risk of severe disease after infection, while vaccination of seropositive subjects results in boosting the immune response, producing a protection comparable with the one obtained in individuals who has had two natural infections (200).

The most severe adverse effect after vaccination was registered when a formalin-inactivated vaccine against RSV produced an increase of severe illness in vaccinated infant (hospitalization: 80% RSV vaccinated *vs.* 5% vaccinated against parainfluenza) and two deaths (201). Afterward, a role for the Th2 response was hypothesized in generating the RSV-mediated ADE (202), and it was demonstrated that the formalin-inactivated virus produced ADE in monkeys (203), suggesting that the carbonyl groups on formaldehyde-inactivated RSV were responsible for the Th2 response in mice (204). Moreover, the observation that formalin inactivation produced an alteration of antigens, leading to the production of non-NAbs, whose avidity did not mature, and the activation of complement were also reported for a measles vaccine (205). The low-avidity non-NAbs are produced in absence of TLR activation (and affinity maturation), and they trigger complement activation (206), enhancing viral infection. To induce potent NAbs, the TLR activation has been obtained using a Th1-polarizing adjuvant (207), in association with the candidate vaccine exposing the epitopes of interest.

Antibody-dependent enhancement has been reported also in many studies focusing on the development of SARS and MERS vaccines, demonstrating that vaccination with the whole S glycoprotein can increase the susceptibility to viral infection with a mechanism not linked to the virus receptor expression on the host cells (208), and especially when antibodies are induced with low titer (209). While for many flaviviruses the mechanism of ADE has been explained through evidences that antibodies developed during a primary infection can enhance entry of a heterologous virus *via* Fc-receptor during a secondary infection, for MERS-CoV and SARS-CoV, it has also been proposed that NAbs that strongly bind the RBD region of the S surface protein can induce conformational changes that enhance the virus entry *via* canonical viral-receptor-dependent pathways, mimicking viral receptor binding (210, 211), and antibodies targeting a specific region of the S protein enhanced the viral infection in a SARS model of NHPs (212). The high sequence homology and the similarity in structure shared among SARS-CoV, MERS-CoV, and SARS-CoV2 S glycoproteins raises reasonable concerns about the development of COVID-19 vaccines based on the S protein.

## Other Options for Pandemic Containment

In potential pandemic settings, the clinical development of vaccines is the main aim. However, apart from technical reasons, the vaccine production might be delayed also for economic considerations and safety issues. Other strategies may be based on self-disseminating vaccines and induction of trained immunity.

To control zoonosis, the formulation of self-disseminating vaccines acts at the level of animal, insect, or environmental reservoir, to directly interfere within the animal-to-human transmission (213). They are essentially based on replicating viral vectors engineered to express the disease antigen and to target a certain animal population (214). Global vaccination of animals could be achieved to effectively contain an emerging pathogen within the wildlife reservoir, avoiding its global spread. Feasibility concerns, costs, and safety issues should be considered when using this strategy to control reservoirs linked to the emergence of high-risk pathogens. In addition, which animal pathogen will cause a human disease is generally unpredictable. It is interesting to underline that a vaccination of great apes with an engineered specific CMV-based vector has been proposed as a strategy to potentially interrupt (or at least decrease) the zoonotic transmission of Ebola virus to humans, being able to protect animals from the lethal viral challenge (213, 215).

Trained immunity-based vaccines (TIbV) might be formulated to stimulate broader anti-infectious responses compared with conventional vaccines for their capacity to increase innate immunity and enhance adaptive responses (216). This strategy exploits the ability of innate immune cells (monocytes, macrophages, NK cells) to undergo extensive metabolic and epigenetic reprogramming, following certain vaccinations or infections, and to become primed for a quite long period of time to respond more potently to autologous or heterologous re-infection, mounting the so-called “innate immune memory.” Triggering of pattern recognition receptors (PRRs) by microbial effector stimuli results in increased production of pro-inflammatory cytokines and/or reactive oxygen species, and in enhanced immune responses, regardless the primary stimulation (217).

Many infectious stimuli are considered potent activators of trained innate immunity, including β-glucan and chitin (components of fungal cell wall), LPS (a component of the cell wall of Gram-negative bacteria), and the Bacille Calmette-Guérin (BCG) vaccine (218). Thus, TIbV should contain pathogen-associated molecular patterns (PAMPs) to target PRRs and subsequently induce trained immune cells. BCG vaccine, VACV, and live attenuated influenza vaccines, together with immunostimulants, could be ascribed to this category of vaccines (216). It is worth mentioning that a whole-cell killed bacterial vaccine might have played a role in preventing pneumonia and mortality during the 1918 Influenza pandemic (219). Recently, a work by Berg and colleagues showed that BCG vaccination is associated with the flattening of the curve in the spread of COVID-19, suggesting that BCV vaccine might serve as a protective factor against the disease (220). However, it should also be noted that an enhanced immune response mediated by reprogrammed immune cells could contribute to the development or maintenance of inflammatory, neuroinflammatory, and chronic metabolic disorders (221). The phenomenon of “trained immunity” occurring in the brain is known as microglial priming. Exposure of primed microglial cells to a second stimuli can cause an augmented inflammatory response, leading to neuroinflammation and production of neurotoxic molecules. The hyperglycemia condition that characterizes type 2 diabetes could long term affect the cellular metabolism of monocytes and macrophages, leading to increased cytokine production and subsequent diabetes complications, including atherosclerosis. An augmented activation of innate cells may also result in the induction and maintenance of chronic inflammatory disorders, including rheumatoid arthritis, systemic lupus erythematosus, multiple sclerosis, or sarcoidosis (221).

## Conclusion

The COVID-19 pandemic experience, combined with the previous viral disease outbreaks, should give blueprints for rapidly responding to the emergence of high-risk pathogens in the future.

It is a common belief that vaccines would be the only means of providing long-term immunity and preventing viral diseases. Despite the great progress made in vaccine research, we are still unable to produce successful vaccines in a timely manner. Human trials take a long time and given a huge list of vaccine candidates, it is hard to choose the most promising one. While the WHO proposed a Solidarity Vaccine trial to test all the candidates in rolling trial until they fail, to increase the chances of succeeding, some vaccine stakeholders are considering extreme alternatives for emergency use: intentionally infect young healthy volunteers at low risk in controlled “human challenge trials” to define which vaccine will work (222). Although these approaches are already used for studying Influenza (223) and Dengue diseases (224), it is hard to ethically accept this option without a validated therapy. Vaccines go through regulatory pathways before the final approval and licensure. In epidemic or pandemic settings, we need to carefully develop a vaccine, as quickly as possible, that adequately proved to be safe and effective (225).

Scientists need to fill the gaps in understanding the epidemiology of novel viruses, to identify potential zoonotic reservoirs or spill-over hosts, and the way of transmission of pathogens. Once the pathogen is identified, preclinical models need to be developed to study virus–host interactions and early test vaccine candidates, defining the immune correlates of protection. Pathogen-specific epitopes need to be identified to guide structure-based vaccines that will elicit protective antibodies, minimizing the induction of non- or weakly NAbs that would promote ADE of viral infection (226). Moreover, data sharing and collaboration among academia, government, and companies will be essential to coordinate a strategic approach in face of next pandemic threats (227).

## Author Contributions

All authors equally contributed to this work and read and approved the final manuscript.

## Conflict of Interest

The authors declare that the research was conducted in the absence of any commercial or financial relationships that could be construed as a potential conflict of interest.
